# *Panax* *notoginseng* extract and total saponin suppress diet-induced obesity and endoplasmic reticulum stress in epididymal white adipose tissue in mice

**DOI:** 10.1186/s13020-022-00629-0

**Published:** 2022-06-20

**Authors:** Yi Tan, Xutao Zhang, Yan Zhou, Lingchao Miao, Baojun Xu, Haroon Khan, Yitao Wang, Hua Yu, Wai San Cheang

**Affiliations:** 1 State Key Laboratory of Quality Research in Chinese Medicine, Institute of Chinese Medical Sciences, University of Macau, Macao SAR, China; 2 Food Science and Technology Program, BNU-HKBU United International College, Guangdong 519087 Zhuhai, China; 3 Department of Pharmacy, Abdul Wali Khan University, Mardan, 23200 Pakistan

**Keywords:** *Panaxnotoginseng*, Saponins, Endoplasmic reticulum stress, Obesity, White adipose tissue

## Abstract

**Background:**

Investigation on protective effects of *Panax notoginseng* against obesity and its related mechanisms is incomplete. Present study aimed to investigate the potential anti-obesity effect of the total saponins (PNS) and ethanolic extract of *P. notoginseng* (PNE).

**Methods:**

Six-week-old male C57BL/6J mice received 45% kcal fat diet for 12 weeks to induce obesity. Oral administration of PNS and PNE at 20 mg/kg/day was applied for the last 4 weeks in the obese mice. Lipid profile was determined by ELISA. Histological examination was performed in liver and fat tissues. Protein levels were measured by Western blot.

**Results:**

PNS and PNE did not cause weight loss. PNE but not PNS decreased the mass of epididymal and retroperitoneal white adipose tissue, accompanied by a reduction in adipocyte hypertrophy. PNS and PNE improved lipid profile by reducing the concentrations of triglyceride, total cholesterol and low-density lipoprotein cholesterol in plasma or liver samples. PNS and PNE also relieved fatty liver in obese mice. PNS and PNE inhibited expression and phosphorylation of endoplasmic reticulum (ER) stress-responsive proteins in hypertrophic adipose tissue.

**Conclusions:**

PNS and PNE can regulate ER stress-mediated apoptosis and inflammation to alleviate obesity.

## Background

Obesity is a growing health crisis worldwide, particularly for developing countries. According to data from the World Health Organization, over 4 million people die each year from being overweight or obese. Obesity defined as abnormal of excessive fat accumulation plays a significant role in the occurrence of many deadly diseases such as cardiovascular diseases, type 2 diabetes and cancers [1–3]. Importantly, adipose tissue, an inert tissue that stores energy in the form of lipids [4], is considered as a metabolic and endocrine organ to modulate immune response, lipid metabolism and other critical biological processes [5]. In obese individuals, excessive fat accumulation induces chronic local inflammation and cell necrosis. This event is characterized by crown-like structure that is formed by the recruitment of macrophages in adipose tissue histologically [6, 7]. Furthermore, adipocytes in obesity are subjected to a variety of stresses and cell dysfunction [8]. Overconsumption of food or excessive lipid uptake causes other non-adipose tissue to suffer lipotoxicity with ectopic lipid accumulation, thereby developing fatty liver, atherosclerosis, and so on [9, 10].

Unfolded protein response (UPR) is a self-protective stress response to reduce the accumulation of unfolded or misfolded proteins in endoplasmic reticulum (ER) [11]. However, when the steady internal state cannot be maintained in ER for prolonged time, ER stress will trigger cell death [12]. Previous studies have strongly supported that chronic activation of ER stress contributes to the development of insulin resistance, lipid metabolism disorders, and chronic inflammation in adipocytes in obesity [13–15]. ER homeostasis play a crucial regulatory role for normal cellular function and survival; and ER stress interplays with multiple perturbations such as inflammation, oxidative stress, and apoptosis in pathological conditions, as for example, in obesity and cardiovascular diseases [16, 17].

*Panax notoginseng* (Burk) F.H. Chen (Sanqi in Chinese) is a member of the Araliaceae family and is a well-known traditional Chinese herb with increasing popularity in the West. In traditional Chinese medicine, *P. notoginseng* has the effects of promoting blood circulation, dissolving stasis, stopping bleeding, reducing swelling, and relieving pain [18]. *P. notoginseng* contains various ingredients including saponins, flavonoids, and so on; whereas *P. notoginseng* saponins (PNS) are well recognized as the major bioactive components for multiple health benefits. Of note, these components have positive effects to combat against cardiovascular diseases and metabolic disorders. PNS can effectively enhance glucose uptake to improve glucose metabolism in *ob/ob* mice and high-fat diet (HFD)-induced obese mice through 5′ adenosine monophosphate kinase (AMPK) activation [19]. Besides, PNS can attenuate coronary heart disease and atherosclerosis [18, 20]. PNS are also shown to regulate lipid metabolism with anti-obesity effects as reported in recent studies, reducing the volume of adipose tissue and mitigating hyperlipidemia through regulation of signaling pathways like leptin, AMPK and peroxisome proliferator-activated receptor (PPAR) [21, 22].

Previous studies have proved that *P. notoginseng* can regulate lipid metabolism and exert anti-obesity effects in addition to the treatment of type 2 diabetes and cardiovascular diseases; nevertheless, pharmacological studies mostly focus on PNS or monomer notoginsenosides. In addition, there are limited studies of *P. notoginseng* on regulating obesity-related ER stress. In present study, we aimed at evaluating the anti-obesity effects of the ethanolic extract of *P. notoginseng* (PNE) to compare with PNS in HFD-induced obese mice and exploring the underlying mechanism. We considered ER stress as a target and thus the regulation of *P. notoginseng* on ER stress in epididymal white adipose tissue (eWAT) was examined.

## Methods

### Extraction of *Panaxnotoginseng* and identification of ethanolic extract (PNE) and total saponins of *Panaxnotoginseng* (PNS)

Extraction of *P. notoginseng* was prepared and quantitative analysis of PNE and PNS was performed as stated in our previous article [23]. In brief, the dried *P. notoginseng* powder was extracted with 95% ethanol and the ethanol was removed by rotary evaporation. Finally, the extract was freeze-dried to obtain PNE. PNS was obtained from Yunnan Yunke Pharmaceutical Co. Ltd. (China). The component analysis of PNS and PNE was determined by Waters ACQUITY-UPLC CLASS system (Waters Corp., USA) with an ACQUITY UPLC BEH phenyl column (150 mm × 2.1 mm, 1.7 μm) maintained at 45 °C to quantify the contents of notoginsenoside R1, ginsenoside Rb1, ginsenoside Re, ginsenoside Rg1, and ginsenoside Rd.

### Diet-induced obese model and herbal treatments

Male C57BL/6J mice were maintained in a temperature-controlled conditions (22–24 °C) with a 12-h light and 12-h dark cycle and were fed with 45 kcal% fat diet (D12451, Research diets, Sysebio, China) at 6 weeks old for 12 weeks. The obese mice were administered with water (vehicle), PNS, or PNE at 20 mg/kg body weight by oral gavage for next four weeks. Mice of control group were fed with standard-chow diet and administered with vehicle. The procedures for care and use of animals were approved by the Animal Research Ethics Committee, University of Macau. Mouse body weight and food intake were measured before and after the experiments.

### Biochemical analysis in plasma and liver

Mice were sacrificed by CO_2_ asphyxiation. Blood samples were drawn from the inferior vena cava and collected in heparin-coated microcentrifuge tubes. Plasma was separated by centrifugation at 3000 rpm at 4 °C for 10 min. Whole liver was isolated from each mouse and weighed. Plasma and liver samples were kept at − 80 ºC until further assay. The levels of total cholesterol and triglyceride in plasma and liver were tested with enzymatic methods by cholesterol assay kit and triglyceride assay kit respectively (Stanbio Laboratory, USA) according to the manufacturer’s instructions. The levels of high-density lipoprotein cholesterol (HDL-C) and low-density lipoprotein cholesterol (LDL-C) were measured using the HDL-C and LDL-C assay kits (Nanjing Jiancheng Bioengineering Institute, China). Plasma levels of alanine aminotransferase (ALT) and aspartate aminotransferase (AST) were determined by ALT and AST assay kits (Nanjing Jiancheng Bioengineering Institute).

### Histological examination and immunohistochemistry

Epididymal white adipose tissue (eWAT) and liver were fixed in 4% paraformaldehyde, embedded in paraffin wax and sliced into Sect. (4 μm). After staining by hematoxylin and eosin (H&E), eWAT and liver sections were observed by light microscope. The images at 200 × magnification were acquired to evaluate histopathological changes by Image J software (National Institutes of Health, USA), determining the number and cell size of adipocytes. The result was calculated as following: the mean cell size = the total area of the cells ÷ the number of the cells. Hepatic sections were stained with Oil Red O and the areas of Oil Red O staining in captured images were determined using Image-Pro Plus software (Media Cybernetics, USA). F4/80 and CD68 antibodies was applied to assess the presence of macrophage infiltration in eWAT and liver respectively (Servicebio, China). Apoptosis in adipose tissue sections was evaluated by terminal deoxynucleotidyl transferase dUTP nick end labeling (TUNEL) staining (Servicebio, China). The images at 200 × or 400 × magnification were acquired to evaluate histopathological changes.

### Western blot assay

Liver and eWAT collected from mice were snap frozen in liquid nitrogen and were subsequently homogenized with RIPA solution (Beyotime, China) containing PhosSTOP and cOmplete Protease Inhibitor Cocktail (Roche, Germany) on ice. The supernatants were collected after centrifugation at 15,000 rpm for 30 min at 4 °C and the total protein contents were measured by BCA Protein assay kit (Beyotime). Equal amounts of proteins (15 µg) were separated by 8–10% SDS-PAGE gels and electrotransferred onto PVDF membrane (Millipore, USA). All materials for SDS–PAGE was acquired from Bio-Rad (USA). After blocking at room temperature with 5% non-fat milk powder (Bio-Rad) which dissolved in Tris buffered saline Tween (TBST) for 2 h, the membranes were incubated overnight with the appropriate primary antibodies at 4 °C followed by incubation with the appropriate secondary antibodies for 2 h at room temperature. Specific binding sites were detected by enhanced chemiluminescence detection solutions (Thermo Fisher, USA) and ChemiDoc MP Imaging System (Bio-Rad). Image Lab (Bio-Rad) was used to quantify the target protein expressions. The primary antibodies against GAPDH, SAPK/JNK, p-SAPK/JNK (Thr183/Tyr185), CHOP, GRP78, p38, p-p38 (Thr180/Tyr182), and caspase-3 were obtained from Cell Signaling Technology (USA); ATF6 was supplied by Abcam (UK); and GRP78 was acquired from Santa Cruz Biotechnology (USA). The secondary anti-rabbit antibodies and anti-mouse antibodies were obtained from Cell Signaling Technology.

### Statistical analysis

All data were presented as mean ± S.E.M from three or more independent experiments. Differences between groups were analyzed using one-way analysis of variance (ANOVA) by GraphPad Prism (USA). P values < 0.05 were considered statistically significant difference.

## Results

### Effects of PNS and PNE on plasma lipid profile and fat mass in obese mice

**Fig. 1:**
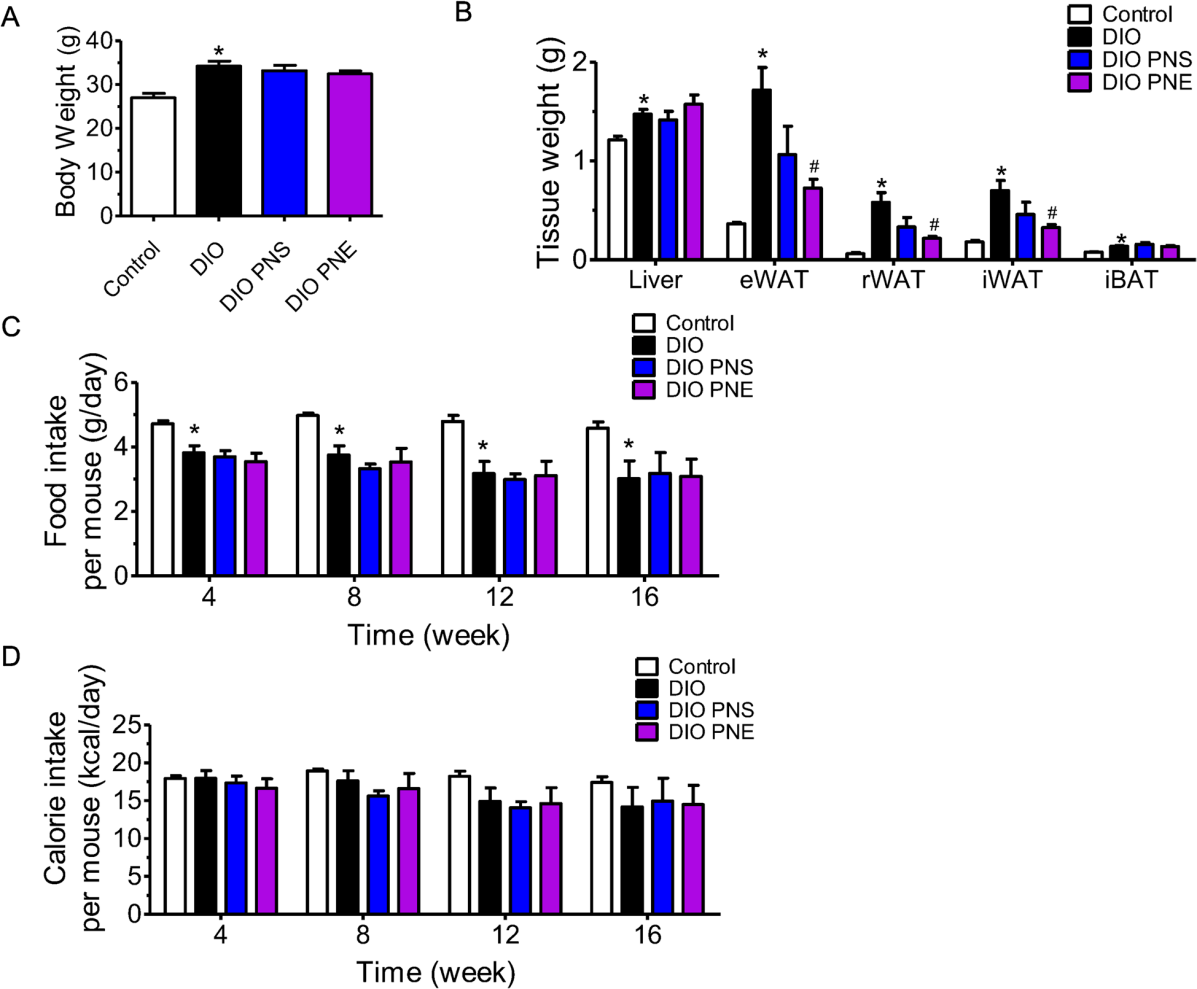
PNS and PNE reduce fat mass. The effects of PNS and PNE treatment (20 mg/kg/day, 4 weeks) on the body weight (**A**), tissue weight (**B**), daily food intake (**C**) and calorie intake (**D**) in DIO mice. Control mice were fed with normal chow diet (3.8 kcal/g) while the other groups were fed with 45% kcal% high-fat diet (4.7 kcal/g). Values are the means ± SEM (n = 6); *p < 0.05, DIO vs. Control; ^#^p < 0.05, PNS vs. DIO and PNE vs. DIO

The obesity model was successfully constructed as the body weight in diet-induced obese (DIO) mice was 20% greater than chow diet-fed mice at the end of 16 weeks, i.e. 34.23 ± 1.15 g and 26.98 ± 1.04 g respectively. Likewise, tissue weights including liver, epididymal white adipose tissue (eWAT), retroperitoneal white adipose tissue (rWAT), interscapular white adipose tissue (iWAT) and interscapular brown adipose tissue (iBAT) were remarkably increased when compared to the control lean mice (Fig. 1A and B). Oral administration with PNS and PNE at 20 mg/kg/day for 4 weeks did not alter body weight or liver weight in diet-induced obese (DIO) mice. For the visceral white adipose tissue tissues, chronic treatment of PNS showed slight but insignificant diminishment of eWAT, rWAT and iWAT whilst PNE had more potent effects to reduce the fat mass in eWAT, rWAT and iWAT. Both of them had no effect on the weights of interscapular brown adipose tissue (iBAT), as well as food and calorie intake (Fig. 1C and D).

**Fig. 2:**
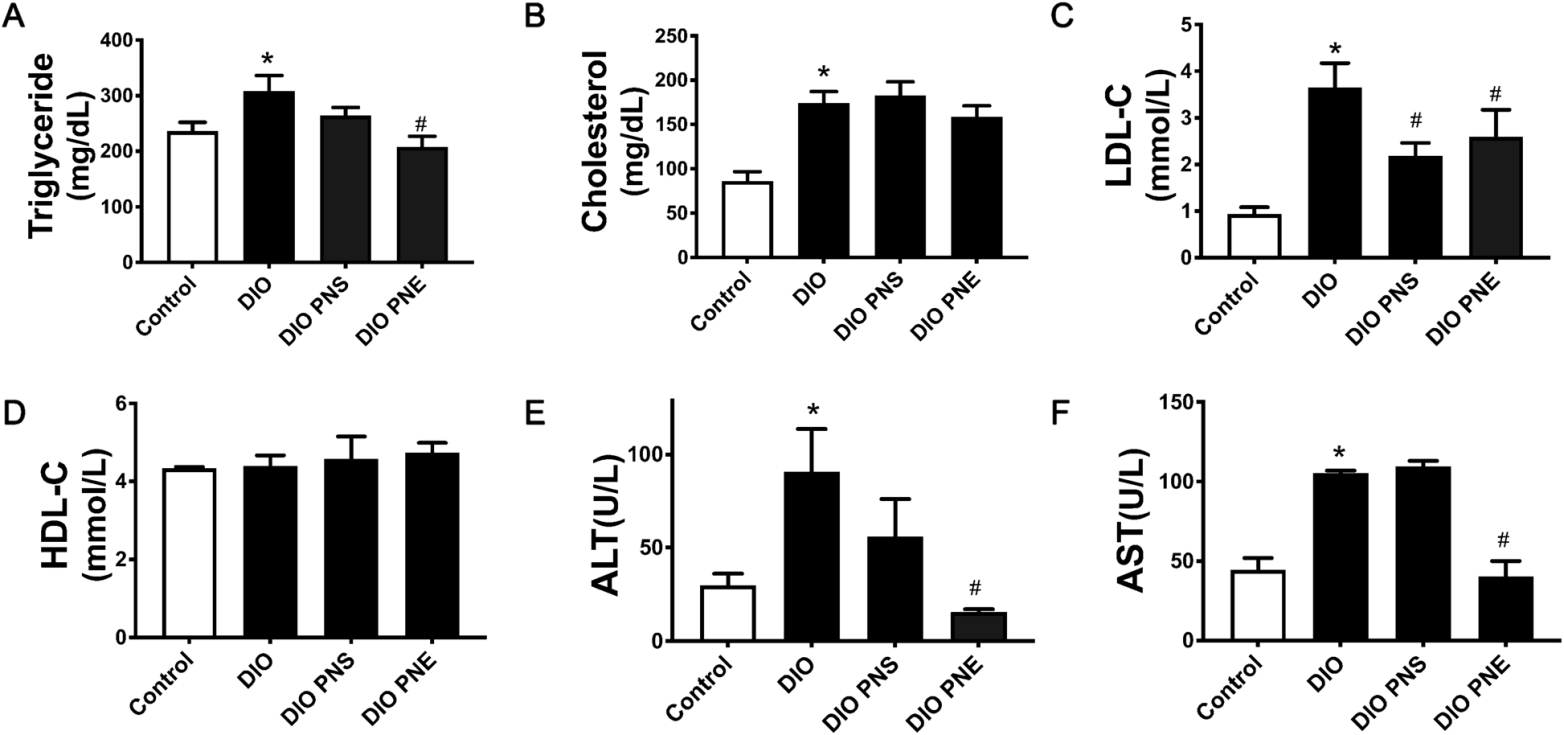
PNS and PNE improve lipid profile. The effects of PNS and PNE treatment (20 mg/kg/day, 4 weeks) on plasma levels of triglyceride (**A**), total cholesterol (**B**), LDL-C (**C**), HDL-C (**D**), ALT (**E**) and AST (**F**) in DIO mice. Values are the means ± SEM (n = 6); *p < 0.05, DIO vs. Control; ^#^p < 0.05, PNS vs. DIO and PNE vs. DIO

To examine the improvement of PNS and PNE treatments in metabolic disturbance, several important biochemical indicators in plasma were tested. Comparing with the control mice, chronic intake of high-fat diet resulted in elevations in the plasma levels of triglyceride, total cholesterol and LDL-C (Fig. 2A–C), whereas the level of HDL-C was not affected (Fig. 2D). PNE treatment was effective to decrease plasma triglyceride and LDL-C concentrations relative to the obesity model group but PNS only reduced plasma LDL-C level. On the other hand, the plasma ALT and AST contents in obese mice were increased as compared with control mice, indicating possible liver function in obesity (Fig. 2E and F). PNE treated group significantly decreased both ALT and AST levels in plasma but PNS did not exert remarkable effect.

### Effects of PNS and PNE on fatty liver

**Fig. 3:**
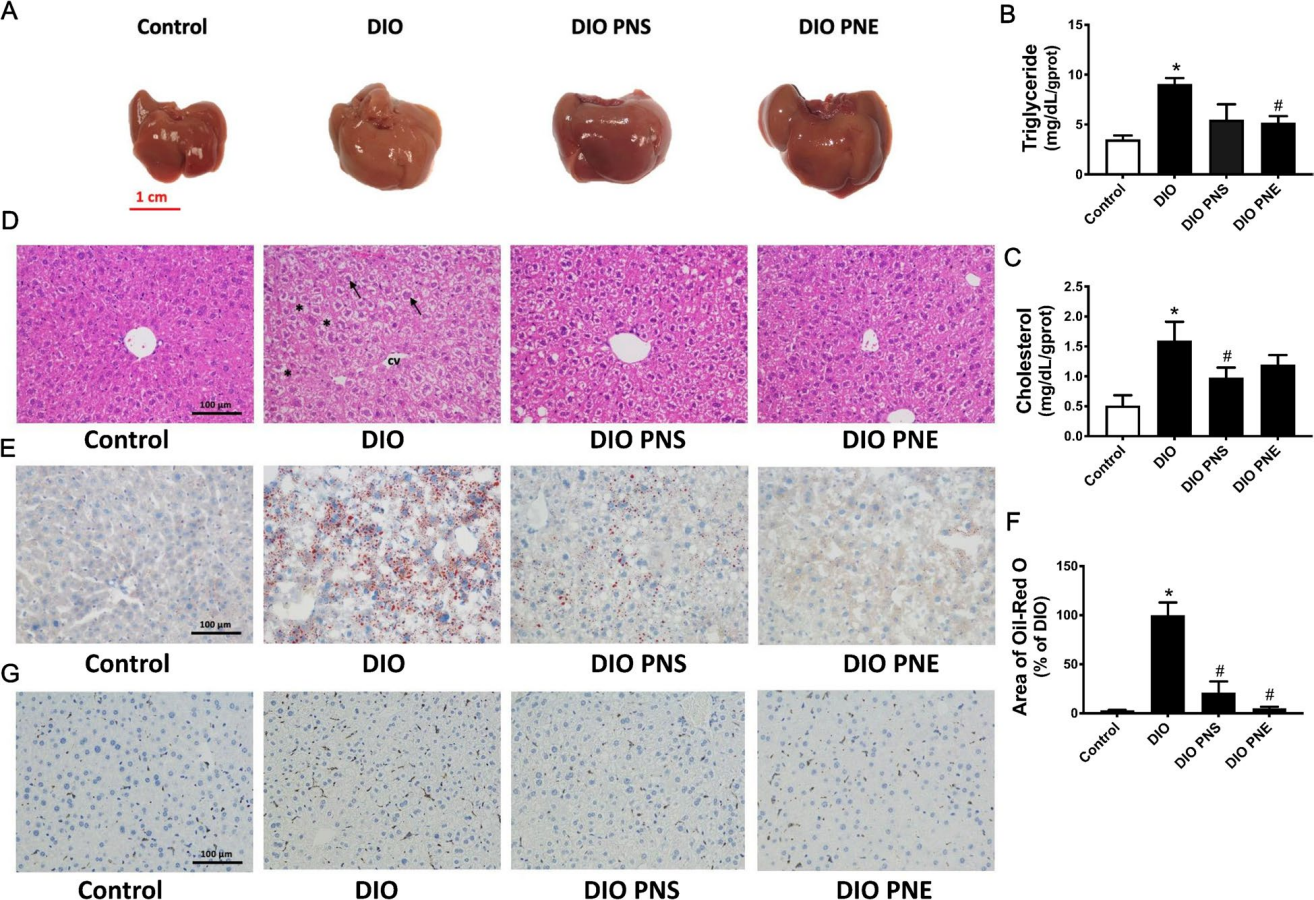
PNS and PNE relieve fatty liver in obesity. Appearance of the liver (**A**). Hepatic levels of triglyceride (**B**) and total cholesterol (**C**). Representative images of H&E (**D**), oil-red O staining (**E**) and CD68 expression (**G**) of the liver histological sections. Relative area of oil-red O staining in liver (**F**). Values are the means ± SEM (n = 6); *p < 0.05, DIO vs. Control; ^#^p < 0.05, PNS vs. DIO and PNE vs. DIO. Hepatocytes (arrow); Vacuolization of cytoplasm (*); central vein (cv)

Liver is a target organ for lipid metabolism, so we examined whether PNS and PNE treatments can affect hepatic morphology and fat accumulation in obese mice. In consistent with the above data on liver weight, livers were enlarged in obesity (Fig. 3A). Moreover, the livers in DIO mice appeared yellowish red, unlike the bright red color observed in the livers of lean mice. PNS and PNE did not reduce the size of liver but reversed the color to a comparable extent as the control. Hepatic triglyceride and total cholesterol levels were increased in DIO mice which can be reversed by PNE and PNS respectively (Fig. 3B and C). The liver segments from normal mice showed regular structure of hepatocyte and hepatic lobule. The cells are compactly arranged and its structure is clear and intact (Fig. 3D). In contrast, the liver cells in the vehicle-treated obese mice were cloudy swelling and loosely arranged and vacuolization of cytoplasm was found. These histopathological manifestations of liver tissue were reversed by the 4 week-treatment of PNS and PNE. The results of oil red staining showed an increased number of intracellular lipid droplets in DIO mice which was suppressed by both PNS and PNE treatments (Fig. 3E and F). Immunohistochemical staining with anti-CD68 antibody (brown) showed that PNS and PNE reversed DIO-induced macrophage infiltration in the liver (Fig. 3G). Taken together, these data suggested that PNS and PNE could attenuate hepatic lipid accumulation and liver damage in DIO mice.

### Effects of PNS and PNE on adipocyte size and adipose macrophage infiltration

**Fig. 4:**
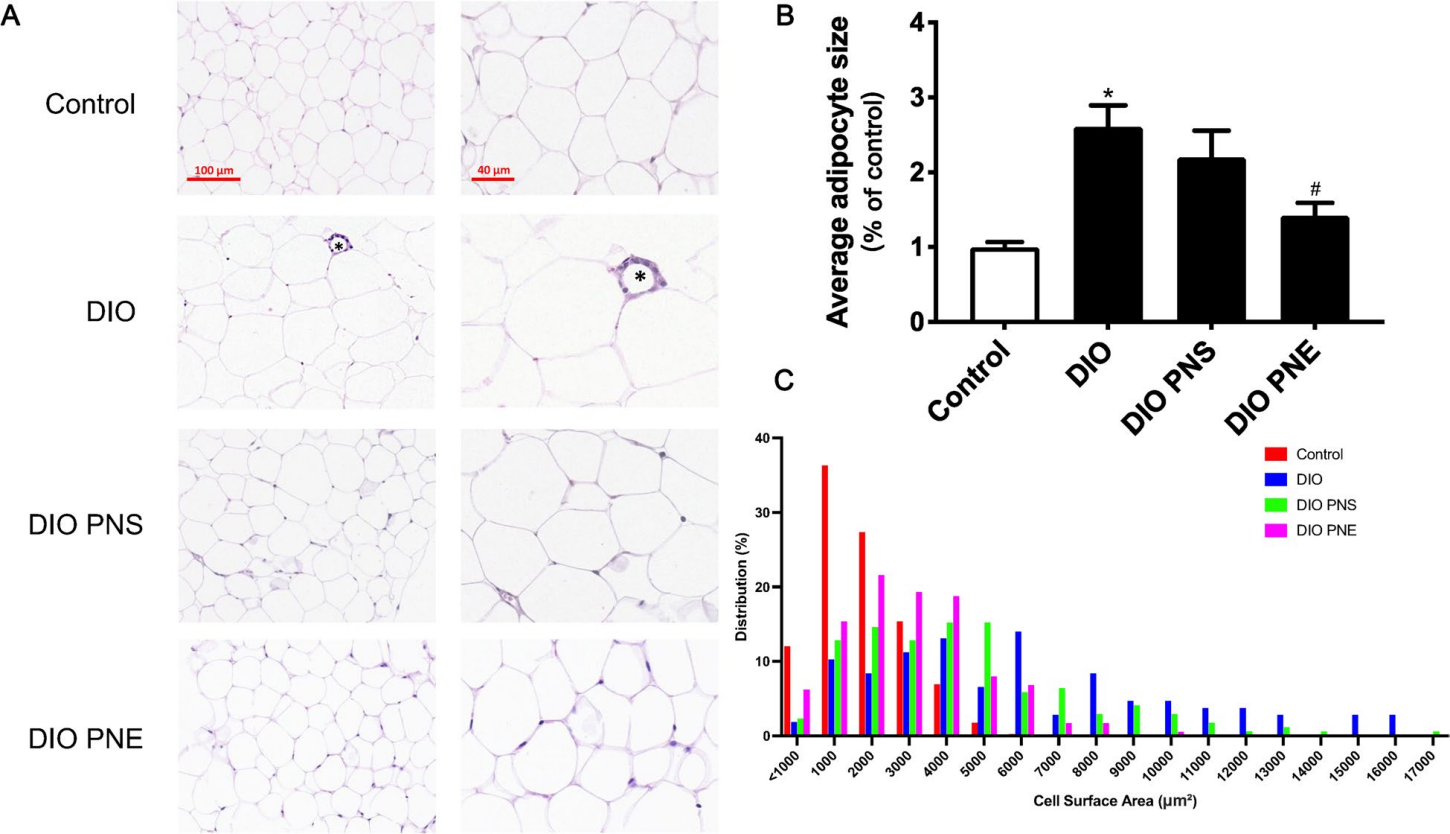
PNS and PNE inhibit adipocyte hypertrophy. Representative images of H&E staining of the eWAT histological sections (**A**). Average size of adipose cells (**B**). The distribution trend of adipocyte size (**C**). Values are the means ± SEM (n = 6); *p < 0.05, DIO vs. Control; ^#^p < 0.05, PNS vs. DIO and PNE vs. DIO. Crown-like structures (*)

**Fig. 5:**
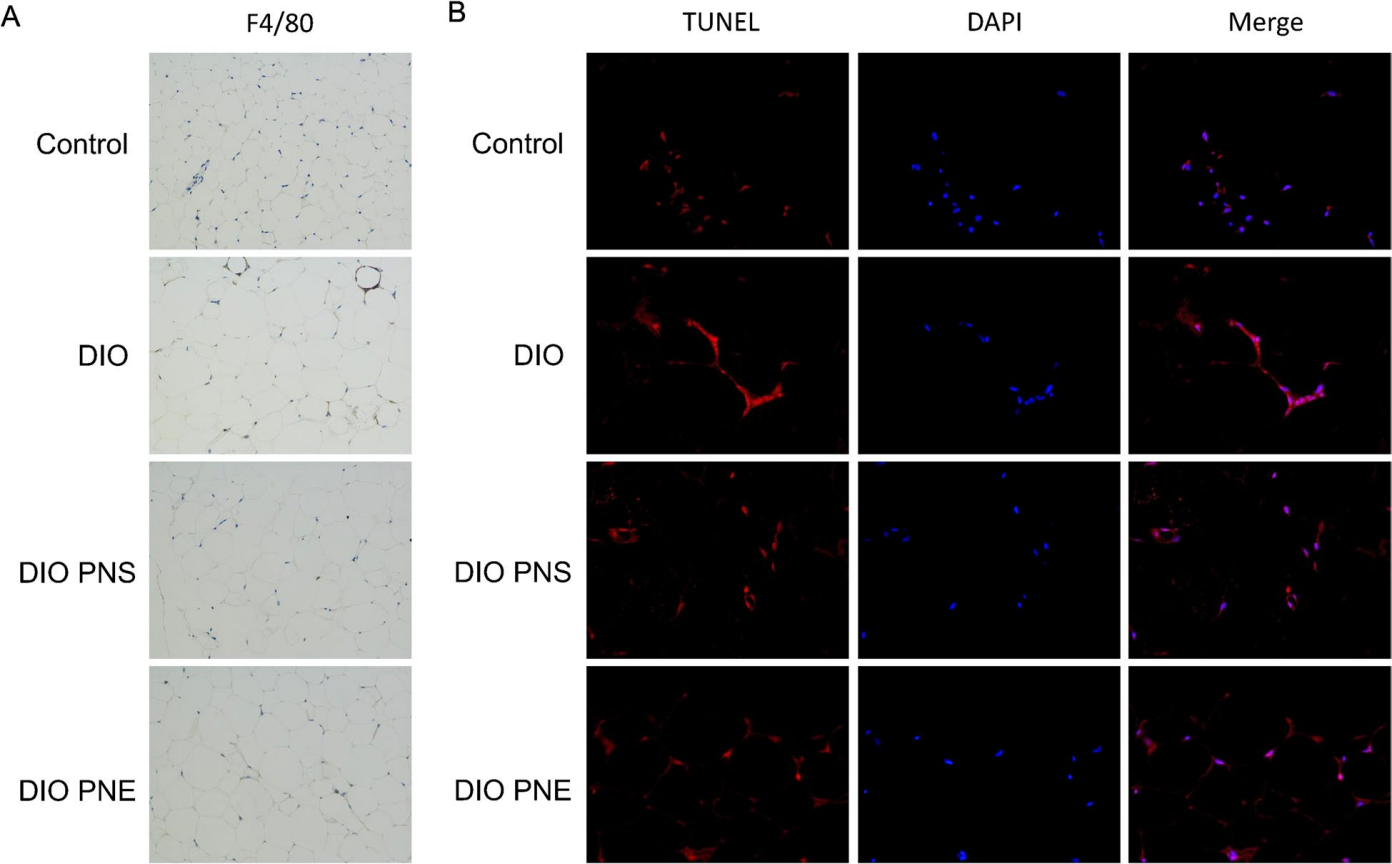
PNS and PNE suppressed inflammation and apoptosis in adipose tissue. F4/80 staining (brown) in the crown-like structure was observed in eWAT of DIO mice under 200 × field (**A**). Adipose tissue apoptosis was determined by TUNEL staining (**B**) while DAPI labels the nucleus to determine the total number of cells in 400x field

As mentioned above, PNS and PNE decreased the fat mass in obese mice. The effects of PNS and PNE in eWAT were explored specifically here. After high-fat feeding, the average adipocytes size of eWAT was significant bigger than control group (Fig. 4A and B). Additionally, the distribution trend of cell size was evaluated. The lean mice had a greater number of small adipocytes (< 2000 μm²) while the distribution shifted to larger size in DIO group in 6000–16,000 μm² (Fig. 4C). In DIO mice, crown-like structures were formed, representing macrophage infiltration in dying or dead adipocytes; but no crown-like structures were found in all eWAT samples in control, PNS or PNE-treated DIO mice. Immunohistochemical staining of eWAT with anti-F4/80 antibody showed that PNS and PNE reversed DIO-triggered macrophage infiltration in the crown-like structure of adipose tissue (Fig. 5A). TUNEL-positive cells was increased in DIO mice and were decreased by both PNS and PNE treatments (Fig. 5B). These results supported that PNS and PNE improved the existential state of adipocytes.

### Effects of PNS and PNE on ER stress-mediated apoptosis and inflammation

**Fig. 6:**
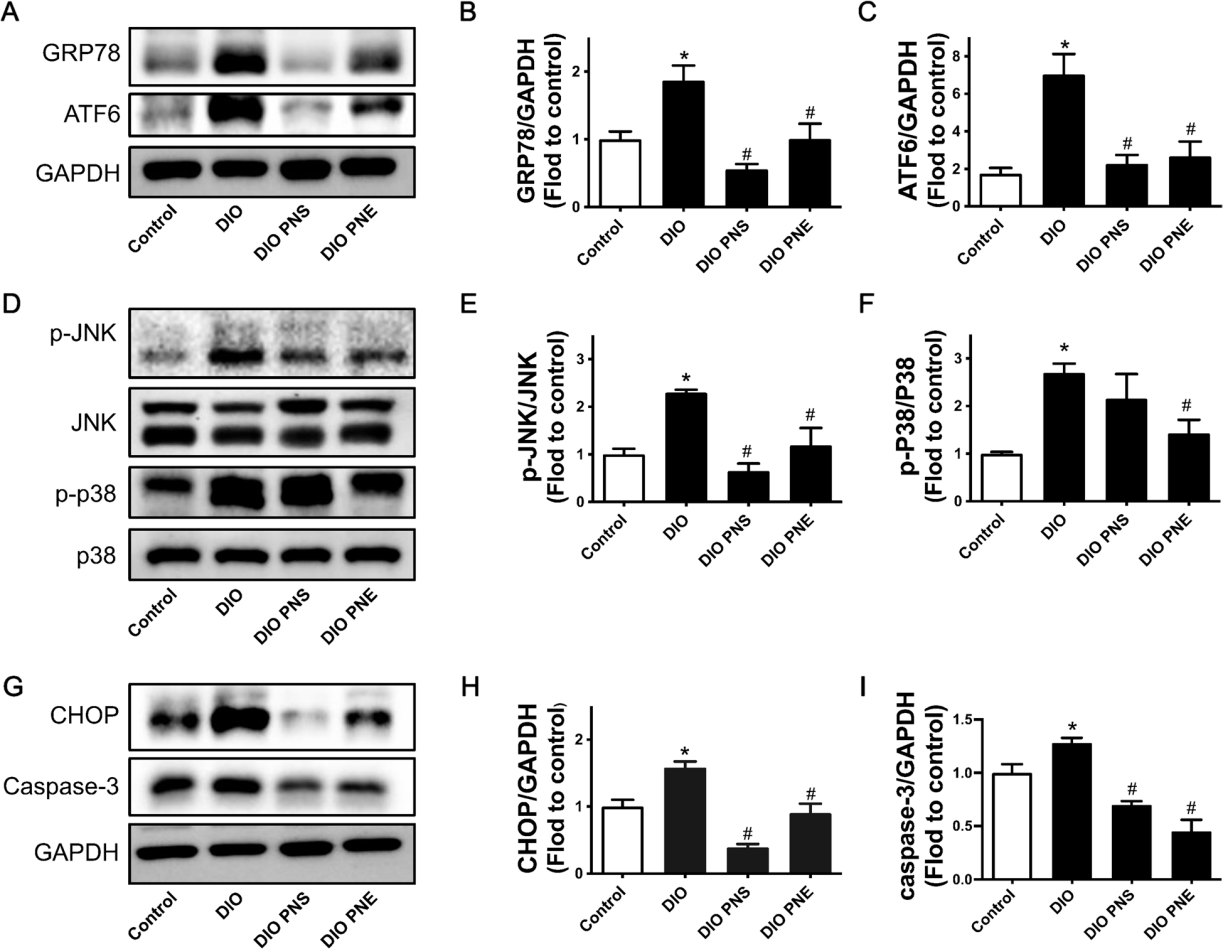
PNS and PNE inhibit ER stress-related signaling pathways. Representative Western blot images (**A**) and summarized data for protein expressions of GRP78 (**B**), and activated ATF6 (**C**) compared to housekeeping protein GAPDH. Representative Western blot images (**D**) and summarized data for phosphorylation of JNK (**E**) and p38 (**F**) compared to its total protein. Representative Western blot images (**G**) and summarized data for protein expressions of CHOP (**H**) and caspase-3 (**I**) in eWAT of DIO mice with different treatments. Values are the means ± SEM (n = 6); *p < 0.05, DIO vs. Control; ^#^p < 0.05, PNS vs. DIO and PNE vs. DIO

In this section, the action mechanism of PNS and PNE improving obesity were explored. First of all, GRP78, ATF6 and JNK, used as indicators of cells undergoing ER stress, were measured by Western blotting. Protein expressions of GRP78 and activated ATF6 (Fig. 6A–C) and phosphorylation of JNK at Thr183/Tyr185 (Fig. 6D and E) were upregulated in eWAT from DIO mice while PNS and PNE reversed these changes, inhibiting ER stress. Like JNK, p38 MAPK modulates inflammation in related to ER stress. Phosphorylation of p38 at Thr180/Tyr182 was downregulated by PNE while PNS showed minor but insignificant effect (Fig. 6D and F). CHOP/caspase-3 is involved in ER stress-induced apoptosis. The upregulation of CHOP and caspase-3 was reduced by PNS and PNE in DIO mice (Fig. 6G–I).

## Discussion

The present study examined the anti-obesity potential of PNS and PNE, and explored the mechanism of obesity-related ER stress and cell apoptosis in DIO mice. Positive results were obtained: (1) chronic treatment of PNE significantly improved the obesity-induced pathological changes such as avoiding an excessive fat accumulation, decreasing blood lipid levels and resisting fatty liver but PNS at the same dosage showed moderate anti-obesity effects; (2) PNS and PNE inhibited obesity-related ER stress and the associated apoptosis and inflammation in eWAT.

In the past decades, the global epidemic of obesity has attracted more and more attention. Metabolic disturbance is aggravated by overnutrition and/or modern sedentary lifestyle and other risk factors consist of environment and genetics [24]. It is well known that obesity causes many health problems, including high blood pressure, fatty liver, diabetes, and increasing the risk of cancers [25]. The most intuitive manifestations of obesity are weight gain and massive accumulation of fat. Expansion of eWAT quickly responds to obesity in body with increasing in adipocyte number and cell size [26]. A number of studies have investigated the effects of *P. notoginseng* or its component on adipocyte by in vitro experiments. In 3T3-L1 cell, PNS and ginsenosides Rb1/Rg1 were shown to inhibit adipogenesis and increase glucose uptake [21, 27, 28]. Therefore, the current study aimed at exploring the anti-obesity properties of PNS and PNE in vivo. In previous study, PNS was found to reduce body weight and fat mass in DIO mice [29]. However, our current data showed that PNS could not avoid these adverse consequences of HFD. These contradictions might be due to the different treatment conditions. In our present study, we treated the mice with a low dosage at 20 mg/kg/day and the HFD was the type with 45% fat; while PNS administered in that previous study was high dosage, 400 and 800 mg/kg/day, and HFD with 60% was used. Importantly, we provided the novel findings that the whole extract PNE was more potent than PNS at the same dosage to reduce the visceral white adipose tissues, eWAT and rWAT, as well as iWAT accompanied by reversal of adipocyte hypertrophy. PNE significantly decreased the fat accumulation (eWAT, rWAT and iWAT) but this did not contribute to significant loss of body weight. The lean mass may play a crucial role. There is a limitation of the current study that the overall lean mass and fat mass in the whole animal were not examined, which require further exploration. Brown adipose tissue is an energy-consuming and heat-producing adipose tissue [30, 31]. PNS and PNE had no influence on food intake and energy consumption as well as the weight of iBAT.

Obesity detrimentally affects lipid metabolism including triglyceride and cholesterol productions. When the storage capacity of adipose tissue reaches saturation, ectopic fat deposition and increased circulating free fatty acids (FFA) will occur due to the extravasation of excess fatty acids from adipocytes, resulting in lipotoxicity to various organs or tissues [32]. Non-alcoholic fatty liver disease can be found during the progression of obesity and typical features are increases in lipid content and liver damage. Herein, both PNS and PNE were able to ameliorate the lipid metabolism and fatty liver: (1) lowering the levels of triglyceride and cholesterol in plasma and liver tissues to different extents; (2) greatly inhibiting hepatic fat accumulation; and (3) diminishing liver function-related indicators ALT and AST. These results are consistent with the recent study on PNS [33]. Amelioration of fatty liver is beneficial to reduce the increase of FFA-induced acyl coenzymes A (CoA) [34], and ultimately prevent the synthesis of endogenous cholesterol [35, 36].

Obesity will lead to cell hypoxia because of limited angiogenesis and excessive adipose tissue, and thereby trigger inflammation and apoptosis [10]. Proinflammatory factors are released in hypertrophic adipose tissue, as for example, tumor necrosis factor-α (TNF-α), interleukin-6 (IL-6) and monocyte chemoattractant protein-1 (MCP-1) [37]. Proinflammatory macrophages are recruited into fat depots by inflammatory factors to form crown-like structures, which are well recognized as the histologic hallmarks of inflammatory and dead adipocytes [38]. The crown-like structures were present in eWAT from DIO mice but were absent in samples from PNS- and PNE-treated groups. This result implies that PNS and PNE reduced cellular inflammation and apoptosis in adipocyte hypertrophy, which were further verified by examining protein expressions of related signaling pathways.

In obesity, adipocyte hypertrophy and massive lipid accumulation are associated with ER stress. ER stress increases macrophage infiltration, triggering inflammation and apoptosis in adipocytes [38, 39]. Upon ER stress, GRP78/BiP, an ER chaperone dissociates from the three ER stress sensors, PERK, IRE1 and ATF6 to activate the downstream signaling cascade. Both JNK and p38 MAPK are known to be downstream targets of IRE1 pathway; and activation of p38/JNK signaling pathway mediates not only apoptosis but also inflammation. All the three pathways ultimately induce the activation of CHOP and caspase-3 to initiate apoptosis [40, 41]. As reported previously, HFD significantly increased the expressions of ER stress-responsive proteins such as CHOP and GRP78 in eWAT [13, 42]. There is also convincing evidence that adipose tissue from DIO mice had enhanced phosphorylation of JNK [43]; and inhibition of JNK activity could reduce adipocyte apoptosis [44]. Moreover, CHOP is linked to inflammation. HFD-induced macrophage infiltration was improved in CHOP^−/−^ mice [45]. Similarly, we found that expressions of GRP78, cleaved (active) ATF6, phosphorylated JNK at Thr183/Tyr185, phosphorylated p38 at Thr180/Tyr182, CHOP and caspase-3 were upregulated in the eWAT from obese mice, revealing the occurrence of ER stress and the associated inflammation and apoptosis. These proteins in eWAT were effectively downregulated by PNS and PNE treatments. Ginsenoside Re exhibits anti-inflammatory role through inhibition of p38/JNK activation in 3T3-L1 cell, as well as relieving the insulin resistance [46]. Ginsenoside Rg3 inhibits ER stress-mediated adipocyte death [47]. In other cell type like cardiomyocytes, PNS [48] and notoginsenoside R1 [49] protect against ER stress-related signaling pathways. The anti-inflammatory potential of PNS has been widely demonstrated [50, 51], and notoginsenoside R1 can suppress p38/JNK pathway to protect PC12 cells from neurotoxicity [52]. In line with the previous studies, we provided the novel findings that not only PNS but also PNE can inhibit ER stress-mediated inflammation and apoptosis in adipocytes, exerting the anti-obesity effect.

## Conclusions

Collectively, our results suggest that high-fat consumption alters ER homeostasis in adipocyte and impairs lipid metabolism. Saponins are always recognized as the major bioactive ingredients of *P. notoginseng.* Interestingly, the whole ethanolic extract also exhibit anti-obesity effects and is more effective than PNS at certain circumstances. PNS and PNE reduce body fat mass and improve lipid distribution in obese mouse model, through regulating the interplay of ER stress, inflammation and apoptosis. The results support the therapeutic potentials of PNS and PNE against obesity and its associated metabolic abnormalities. The advantages of PNE over PNS on lipid metabolism may be attributed to other ingredients apart from saponins. Further investigation is needed to identify the mediator(s) contributing to the differential effects of PNS and PNE.

## Data Availability

Not applicable.

## Abbreviations

ALT: Alanine aminotransferase
AMPK: 5′ adenosine monophosphate kinase
AST: Aspartate aminotransferase
DIO: Diet-induced obese
ER: Endoplasmic reticulum
eWAT: Epididymal white adipose tissue
FFA: Free fatty acids
HDL-C: High-density lipoprotein cholesterol
HFD: High-fat diet
IL-6: Interleukin-6
LDL-C: Low-density lipoprotein cholesterol
MCP-1: Monocyte chemoattractant protein-1
PNE: Ethanolic extract of *Panax* *notoginseng*
PNS: *Panax* *notoginseng* saponins
PPAR: Peroxisome proliferator-activated receptor
rWAT: Retroperitoneal white adipose tissue
TNF-α: Tumor necrosis factor-α
UPR: Unfolded protein response
iWAT: Interscapular white adipose tissue
iBAT: Interscapular brown adipose tissue
